# Dyke-Davidoff-Masson syndrome: a case report

**DOI:** 10.1186/s12883-018-1079-3

**Published:** 2018-05-29

**Authors:** Anna Misyail Abdul Rashid, Mohamad Syafeeq Faeez Md Noh

**Affiliations:** 1 Department of Medicine, Faculty of Medicine and Health Sciences, Universiti Putra Malaysia, Serdang, Malaysia; 2 Department of Imaging, Faculty of Medicine and Health Sciences, Universiti Putra Malaysia, Serdang, Malaysia

**Keywords:** Dyke-Davidoff-Masson syndrome, Computed tomography (CT), Magnetic resonance imaging (MRI)

## Abstract

**Background:**

Dyke-Davidoff-Masson syndrome is a rare condition of unknown frequency resulting from brain injury due to a multitude of causes; especially in early life. Characteristics include cerebral hemiatrophy/hypoplasia, contralateral hemiparesis, seizures, and compensatory osseous hypertrophy.

**Case presentation:**

We present a case of a 13-year-old girl who initially presented with headaches, followed by episodic complex-partial seizures; which was controlled via medication. She also had right sided hemiparesis. Computed tomography (CT) showed evidence of left parieto-temporal infarct with cerebral atrophy. Complementary magnetic resonance imaging (MRI) did not reveal additional information. Workup for young stroke was negative. Upon further evaluation by Neuroradiology, features suggesting Dyke-Davidoff-Masson syndrome were confirmed. Patient has been under Neurology follow up since.

**Conclusions:**

Due to its rarity, Dyke-Davidoff-Masson syndrome may easily be missed by the majority of treating clinicians. Knowledge of its features on imaging enables timely and accurate diagnosis – allowing appropriate management.

## Background

Dyke-Davidoff-Masson syndrome (DDMS) is a rare neurological condition of unknown frequency, with available literature mostly from case reports/series [1–3]. Most affected patients are among the pediatric population. Due to its rarity, it may be misdiagnosed or under-reported by the majority of clinicians. We describe a patient who was initially thought to have young stroke of unknown etiology; eventually diagnosed having DDMS via imaging findings.

## Case presentation

**Fig. 1:**
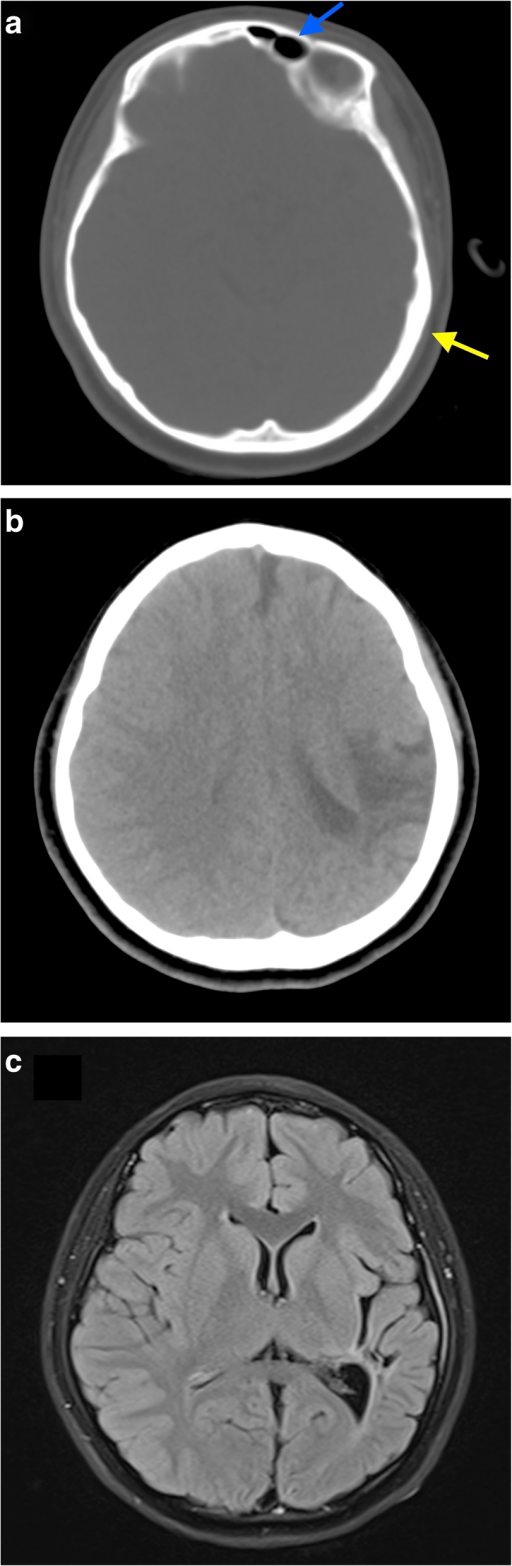
CT (**a**, **b**) and MRI (**c**) images, in axial section showing. **a** CT image in bone window showing hyperpneumatization of the left frontal sinus (blue arrow) with compensatory calvarial thickening (yellow arrow). **b** CT image (non-contrasted) showing the left parieto-temporal infarct. **c** MRI T2 FLAIR image showing left cerebral hemiatrophy, with dilated left lateral ventricle. Focal encephalomalacia and gliotic changes are also noted

A 13-year-old girl presented to us for further management of episodic complex-partial seizures and right hemiparesis. She initially had episodes of headaches starting at the age of 7. Mother noticed that she had accompanying right sided weakness; which prompted medical attention. Initial evaluation revealed power of 4/5 on the right side, with reduced sensation and brisk reflexes. Minimal facial asymmetry was evident. A head CT was done, which revealed left parieto-temporal infarct, with cerebral atrophy. Complementary MRI was of no significant additional value. Workup for young stroke, which included protein S, protein C, anti-thrombin III, anti-phospholipid antibody, anti-cardiolipin antibody, and lupus anticoagulant were negative; she was subsequently put on routine follow up. One year after the initial presentation, her mother noticed new onset of brief moments of the child mumbling followed by blank stare and drowsiness, prior regaining consciousness. She was diagnosed as having complex-partial seizures, and initiated on syrup sodium valproate 200 mg bd. Upon further evaluation of the previous imaging studies, in addition to the left sided infarct, we noticed that the left cerebral hemisphere was universally atrophic with ventricular enlargement. There was also evidence of calvarial thickening on the ipsilateral side, and hyperpneumatization of the left frontal sinus (Fig. 1). A final diagnosis of DDMS was made. Patient has been on regular Neurology follow up since.

## Discussion and conclusions

In 1933, Dyke, Davidoff, and Masson described 9 patients with clinical characteristics of hemiparesis, facial asymmetry, seizures, and mental retardation noted to have pneumatoencephalographic changes on skull radiograph [4]. CT and MRI features of this entity include cerebral hemiatrophy, ipsilateral ventriculomegaly, hyperpneumatization of the sinuses on the affected side, and compensatory calvarial thickening [1, 3, 5]. Affected patients are largely from the paediatric population; however, occurrence in adult patients have been reported [3]. Common causes include congenital anomalies, perinatal hypoxia, intracranial hemorrhage, and infections [1].

Clinically, patients may have seizures, mental retardation, contralateral hemiparesis, and facial asymmetry. Our patient initially had headaches, followed by episodic complex-partial seizures. Birth history did not reveal any hypoxic-ischemic events. There was also no history to suggest intra-uterine/perinatal infection. Only after head CT, an infarct was diagnosed; and patient was treated symptomatically. The MRI that followed did not add any additional information. To the unaccustomed, subtle findings on CT/MRI may easily be missed. We were able to accurately diagnose the imaging features after consulting with Neuroradiology. Understandably, the rarity of this condition makes accurate diagnosis a challenge – as evident in our experience.

Imaging via CT and MRI proves to be of significant value; enabling correct diagnosis and institution of appropriate management. These two imaging modalities are valuable in that they provide cross sectional images, with thin slices, and post processing capabilities. Pertinent imaging features for DDMS include cerebral hemiatrophy/hypoplasia, hyperpneumatization of the paranasal sinuses, and compensatory osseous hypertrophy. These radiological features will become more evident with time, as the patient gets older. There have been reports suggesting calvarial involvement, as in our experience, points to cerebral damage occurring during the intrauterine period or before the age of 3 [6–8]. Our patient, interestingly, did not have the clinical history or features to suggest an early life event which may lead to cerebral damage.

In essence, due to the rarity of this syndrome, it may be easily misdiagnosed by the untrained eye. CT and MRI are powerful imaging modalities to diagnose the pertinent imaging features associated with this syndrome. Knowledge of the clinical presentation, risk factors, and imaging features is therefore indispensable for appropriate patient management.

## Abbreviations

CT: Computed tomography
DDMS: Dyke-Davidoff-Masson syndrome
MRI: Magnetic resonance imaging
